# Mesenchymal Stem Cells: Allogeneic MSC May Be Immunosuppressive but Autologous MSC Are Dysfunctional in Lupus Patients

**DOI:** 10.3389/fcell.2019.00285

**Published:** 2019-11-15

**Authors:** Rui-Juan Cheng, An-Ji Xiong, Yan-Hong Li, Shu-Yue Pan, Qiu-Ping Zhang, Yi Zhao, Yi Liu, Tony N. Marion

**Affiliations:** ^1^Department of Rheumatology and Immunology, West China Hospital, Sichuan University, Chengdu, China; ^2^Department of Rheumatology and Immunology, Nanchong Central Hospital, The Second Clinical Medical College, North Sichuan Medical College, Nanchong, China; ^3^Department of Microbiology, Immunology, and Biochemistry, University of Tennessee Health Science Center, Memphis, TN, United States

**Keywords:** systemic lupus erythematosus, mesenchymal stem cells, dysfunction, senescence, immunoregulatory

## Abstract

Mesenchymal stem cells (MSCs) have a potently immunosuppressive capacity in both innate and adaptive immune responses. Consequently, MSCs transplantation has emerged as a potential beneficial therapy for autoimmune diseases even though the mechanisms underlying the immunomodulatory activity of MSCs is incompletely understood. Transplanted MSCs from healthy individuals with no known history of autoimmune disease are immunosuppressive in systemic lupus erythematosus (SLE) patients and can ameliorate SLE disease symptoms in those same patients. In contrast, autologous MSCs from SLE patients are not immunosuppressive and do not ameliorate disease symptoms. Recent studies have shown that MSCs from SLE patients are dysfunctional in both proliferation and immunoregulation and phenotypically senescent. The senescent phenotype has been attributed to multiple genes and signaling pathways. In this review, we focus on the possible mechanisms for the defective phenotype and function of MSCs from SLE patients and summarize recent research on MSCs in autoimmune diseases.

## Introduction

Systemic autoimmune disease is caused by abnormal immune reactivity and antibody production to self-antigens with subsequent inflammation and damage to host tissues or target organs such as skin, kidney, joints, and muscles (Fanouriakis et al., 2019). Systemic lupus erythematosus (SLE) is a systemic autoimmune disease characterized by aberrant activation of lymphocytes and autoantibody production (Marion and Postlethwaite, 2014). Prominent among the autoantibody specificities in SLE are nuclear antigens including DNA, RNA, ribonuclear proteins, and histones. Pathogenesis in SLE is notoriously heterogeneous and may involve multiple connective tissues, skin, and organ systems. Similarly, the disease heterogeneity complicates diagnosis (Petri et al., 2012). Anti-inflammatory and immunosuppressive drugs have improved survival and prognosis of SLE patients (Bernatsky et al., 2006) and notably in China, according to the statistics of the Chinese SLE Treatment and Research group (CSTAR) (Zhao et al., 2016). Renal disease has the highest standardized mortality ratio in SLE, although patients who fail to respond to conventional therapies or whose disease is accompanied with pulmonary arterial hypertension (PAH) or other serious complications, also have high mortality.

Recently, mesenchymal stem cell (MSC) transplantation has emerged as a promising therapy in refractory SLE patients because of MSCs’ strong immunosuppressive potential (Barbado et al., 2018). MSCs are multipotential, self-replicating stem cells that may differentiate into different mature specialized cells (Weissman and Shizuru, 2008). MSCs can modulate both the adaptive and innate immune system in patients with autoimmune diseases (Munir and McGettrick, 2015), although the intricate details by which MSCs exert their therapeutic effects are not fully understood.

Bone marrow-derived MSCs (BM-MSCs) were effective in clinical trials for steroid-resistant graft-versus-host disease (GVHD), a complication in allogeneic bone marrow transplantation (Le Blanc et al., 2008). The MSCs elicited a suppressive immunoregulatory response that was thought to involve multiple immunosuppressive mechanisms. A 6 years follow-up observational study has provided evidence for the safety and efficacy of allogeneic umbilical cord-derived MSCs transplantation in refractory SLE patients (Wang et al., 2017b). Although a large proportion of the refractory SLE patients attained clinical remission or reduced disease activity after transplantation of allogeneic MSCs from healthy, non-autoimmune individuals, SLE patients benefit little from autologous stem cell transplantation (Barbado et al., 2018), implying that the capabilities of MSCs from SLE patients may be impaired. In this review, we aim to further explore the potential mechanisms that account for the apparent dysfunction of MSCs derived from SLE patients by referring to relevant literature.

## Overview of Mscs

Stem cells are a class of undifferentiated cells in multicellular organisms that are pluripotential and self-replicating. Through unequal cell division, stem cells produce one daughter cell with multiple differentiation potential and one identical daughter cell for self-renewal (Weissman and Shizuru, 2008). There are two major types of stem cells in mammals. One is the embryonic stem cell from the inner cell mass of blastocysts that can form cells from all three embryonic germ layers, endoderm, mesoderm, and ectoderm (Thomson et al., 1998). The other is the adult stem cell, which can selectively replenish dying cells and regenerate damaged tissues, for example hematopoietic stem cells (HSC) in bone marrow and intestinal stem cells in the small intestine (van der Flier and Clevers, 2009).

**FIGURE 1:**
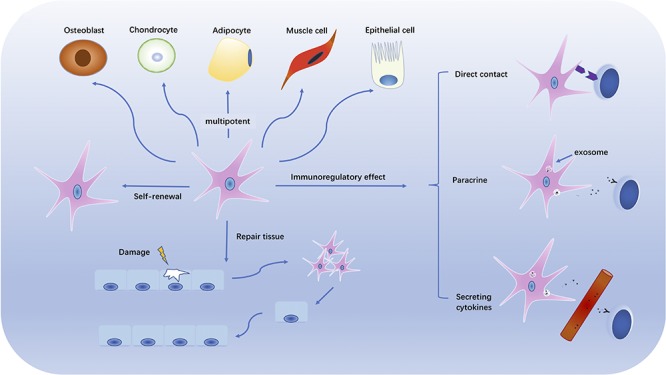
The multipotentiality and immunomodulatory effects of MSCs. The figure illustrates the multitasking capabilities of MSC. Those capabilities include self-renewal, damaged tissue repair, and multipotential differentiation into multiple mesodermal cell types. MSCs also have immunoregulatory function with potential to inhibit or suppress autoimmune and chronic inflammatory reactions by direct cell contact, paracrine release and secretion, and/or cytokine secretion.

In 1976, multipotential stromal precursor cells were identified by Friedenstein et al. (1976) since the bone marrow cells they cultured differentiated into bone-like and chondrocyte-like cells both *in vitro* and *in vivo*. The concept that the multipotential stromal precursor cells, MSC, identified by Friedenstein et al., could be used to therapeutic benefit was raised in 1991 (Caplan, 1991). Bone marrow MSCs can be expanded 10^4^–10^8^-fold *in vitro* with a Hayflick’s limit of 13–25 doublings (Wagner et al., 2008). *In vitro* cultured MSC retain multipotent stromal cell potential and may differentiate into multiple mature cell types from mesodermal lineage such as lipocytes, osteoblasts and chondrocytes. The latter potential is what provides them with potential for regenerative and traumatic medicine (see Figure 1).

The International Society for Cellular Therapy proposed minimal criteria to define human MSC in 2006 (Dominici et al., 2006). Cultured MSC must be plastic-adherent and capable of differentiating into adipocytes, osteoblasts, and chondroblasts *in vitro*. Phenotypically, MSC must express CD73, CD90, and CD105 but none of the hematopoietic differentiation markers CD11b, CD14, CD34, CD45, CD19, CD79α, or HLA-DR. Bone marrow was the first identified and, historically, most frequently utilized source of MSCs. More recently, MSCs have been identified in and isolated from umbilical cord, adipose tissue, molar cells, urine, amniotic fluid, and connective tissue (Dong et al., 2018; Xie and Shen, 2018).

MSCs have aroused widespread interest because they are capable of differentiating into both mesenchymal and non-mesenchymal lineages after isolation from several tissues and *in vitro* expansion. MSCs are considered promising reagents in regenerative medicine and cell-based therapies because of their self-renewal and multilineage potential (Squillaro et al., 2016). The culture environment to which stem cells are exposed is especially relevant for their differentiation. The specific lineages into which naïve MSCs will differentiate and the morphology and phenotypes they will display depend upon differential *in vitro* culture conditions that may vary among individual donors (Engler et al., 2006; Lin et al., 2017; Stojanovic et al., 2018). The proliferative and differentiative capabilities of MSCs decline with donor age and passage number of MSC cultures *in vitro* (Infante and Rodriguez, 2018). To obtain functionally differentiated cells or tissues, stem cells have been cultivated *in vitro* under controlled conditions. The environmental cues (Dinsmore et al., 1996; Liu et al., 2017) that control MSC differentiation include special culture media, various chemical, biological and physical factors, and mechanical stimuli. For instance, osteogenic stimuli such as dexamethasone, ascorbic acid, and β-glycerophosphate can promote the osteogenic differentiation of cultured MSCs. Osteogenic differentiation can be distinguished by the ALP activity, deposition of extracellular calcium, and expression of osteogenic genes. Furthermore, studies revealed that miRNAs and several signaling pathways may affect the regulation of MSC differentiation (Sun X.K. et al., 2017).

## The Immunoregulatory Activities of Mscs

**FIGURE 2:**
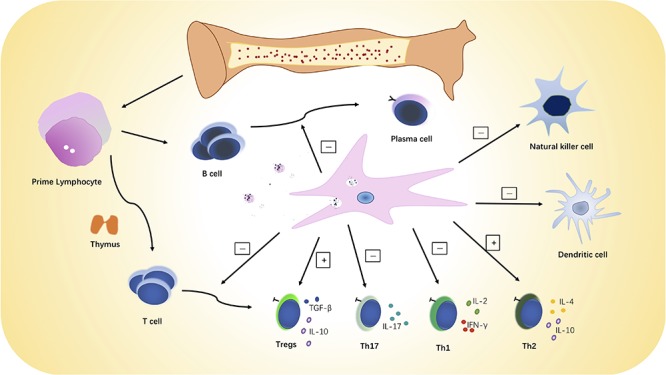
MSC immunoregulatory activities. MSCs can inhibit the proliferation and activation of B and T lymphocytes and NK cells and increase or restore the ratio of Tregs to Th effector cells. MSCs can also promote a switch from pro-inflammatory to anti-inflammatory phenotype and cytokine secretion by T cells, dendritic cells, and natural killer cells.

MSCs have immunoregulatory effects on multiple immune system cells and functions (see Figure 2). MSCs mediate their immunoregulatory effect by secreting soluble factors or directly interacting with a variety of immune effector cells (Gebler et al., 2012), and it should be emphasized that MSCs are not always immunosuppressive. MSCs may have different properties and immunoregulatory effects depending on the inflammatory milieu and disease setting (Djouad et al., 2005; Zhou et al., 2013; Dorraji et al., 2018). MSCs can suppress proliferation of both CD4 + and CD8 + T lymphocytes *in vitro* in a dose-dependent, non-apoptotic-induced manner, and the immunosuppressive properties against T cells varies among different MSC sources (Di Nicola et al., 2002; Castro-Manrreza et al., 2014). Transforming growth factor-β (TGF-β), prostaglandin E2 (PGE2), nitric oxide (NO), and indoleamine 2,3-dioxygenase (IDO) were reported to be involved in the MSC-mediated T cell suppression (Aggarwal and Pittenger, 2005; Groh et al., 2005; Sato et al., 2007; Li W. et al., 2012). MSCs can also exert immunoregulatory effects by release of microvesicles (MVs) (Di Trapani et al., 2016) although several studies have substantiated the *in vitro* superiority 0f MSCs over their MVs for antiproliferation effects on T cells (Gouveia de Andrade et al., 2015; Di Trapani et al., 2016). For that reason, infusion of MVs may not be the ideal substitute for MSCs in affecting immune-mediated disorders where T cells are predominate.

MSCs secrete numerous cytokines, chemokines, and hormones to exert paracrine effects on adjacent immune cells to modulate their proliferation, differentiation, migration, and adhesion functions under injury conditions (Meirelles Lda et al., 2009). Paracrine effects of MSC have been referred to as trophic effects, which can be divided into immunosuppressive, anti-fibrogenic, anti-apoptotic, pro-angiogenic, and pro-mitogenic functions (Caplan and Dennis, 2006). Several chemokine receptors including CXCR5, CCR1, CCR4, CCR7, and CCR10 are expressed on MSCs, which might be involved in their homeostatic and tissue-specific recruitment (Von Luttichau et al., 2005). In addition, MSCs secret PD-L1 and PD-L2, two ligands of the programmed death-1 (PD-1) receptor, that inhibit activation and proliferation of T cells, suppress T cell effector function, and modulate peripheral tolerance (Davies et al., 2017). The ability of MSCs to be therapeutic without engraftment or differentiation into tissue-specific cells may expand their range of clinical applications.

Different subpopulations of T cells are differentially affected by the immunoregulatory potential of MSCs. IFN-γ and TNF-α synergistically enhanced the ability of MSCs to adhere to Th17 *in vitro*, inhibited the differentiation of naïve T cells into Th17, and promoted the expression of Foxp3 so as to facilitate differentiation of Th17 toward regulatory T cells (Tregs) that in turn inhibited CD4^+^ Th effector cell activation (Ghannam et al., 2010). MSCs can inhibit T follicular helper (Tfh) differentiation and IL-21 cytokine production *in vitro* and in autoimmune MRL-MP*^*lpr/lpr*^* mice *in vivo*. The *in vivo* MSCs infusion prolonged life and alleviated lupus nephritis in the autoimmune mice (Yang et al., 2018). Autologous MSCs from two SLE patients were able to inhibit lymphocyte activation *in vitro* and, when respectively infused, increased the circulating Treg cell number *in vivo* but had no effect on disease activity (Carrion et al., 2010). In lupus-prone mice, infusion of BM-MSCs did not significantly affect serum anti-dsDNA autoantibody but did result in improved renal histopathology, including reduced immune complex deposition, glomerular proliferation, and lymphocytic infiltration (Schena et al., 2010), all of which are consistent with a potential role for MSCs in the prevention of glomerular damage. Further, the renoprotective effect has been shown to be partially mediated by paracrine effects in mice (Togel et al., 2005). An imbalance in Th1 and Th2 cytokine profiles is suggested to play an important role in the pathogenesis of GVHD, SLE, and other autoimmune diseases. While it is debatable how MSCs modulate and balance the differentiation of the Th1 and Th2 lymphocyte subpopulations, most research on MSC function in immunosuppression has revealed that MSCs exert an immunomodulatory effect by activating or increasing Treg and Th2 and inhibiting or decreasing proinflammatory Th1 and Th17 cells both *in vivo* and *in vitro* (Ghannam et al., 2010; Luz-Crawford et al., 2013; Campanati et al., 2017; Sun Y. et al., 2017; Yan et al., 2017). Results from a few studies indicate that MSCs showed no significant effects on the proliferation or secretory function of Th1 or Th2 subsets individually but did cause a global decrease in the ratio of Th1/Th2 cells (Lim et al., 2014; Choi et al., 2016). In contrast at least one study in arthritic mice indicated that MSC transplantation promoted accumulation of Th1 (Gonzalo-Gil et al., 2016). Nevertheless, in general, MSCs can inhibit proinflammatory cytokine secretion and reduce Th1/Th2 ratios.

Currently, there are contradictory viewpoints regarding immunomodulatory properties of MSCs on B lymphocyte proliferation and activation (Corcione et al., 2006; Tabera et al., 2008; Schena et al., 2010). Corcione et al. (2006) observed that MSCs induced inhibitory effects on B cell proliferation, antibody secretion, and chemotactic function. MSCs blocked proliferation of B cells in the G0 or G1 phase of the cell cycle, but they were not induced to apoptosis (Corcione et al., 2006; Tabera et al., 2008). Schena et al. (2010) found that MSCs inhibited the proliferation of mature murine splenic B cells in a dose-dependent and cell-to-cell contact-dependent manner but failed to affect B cell differentiation to plasma cells. The immunoregulatory effects of MSCs on B cells are mediated, at least in part, by secreted cytokines. The immunosuppressive activity on B cells *in vivo* maybe partially, if not mostly, due to the inhibition of Th cells by MSCs.

As for innate immune cells, MSCs also play a key role in modulating the maturation and function of dendritic cells (DC) (Zhang et al., 2004), potent antigen presenting cells. MSCs alter cytokine secretion from DC1 and DC2, stimulating a decrease in the secretion of TNF-α by DC1 and an increase in IL-10 from DC2 (Aggarwal and Pittenger, 2005). MSCs have been shown to mediate the polarization of macrophages by skewing macrophages toward the M2 lineage (Cho et al., 2014), an anti-inflammatory macrophage phenotype. In addition, MSCs can also inhibit the proliferation, cytokine secretion, and cytotoxicity of natural killer (NK) cells (Li et al., 2015).

MSCs exhibited two distinct, non-MHC-restricted immunomodulatory functions that depended on the relative numbers of MSCs and local inflammatory conditions. Low numbers of MSCs had less inhibitory effects and sometimes enhanced lymphocyte proliferation, whereas large doses of MSCs always exerted a suppressive effect (Le Blanc et al., 2003; Bocelli-Tyndall et al., 2009). MSCs provided no clinical amelioration in murine collagen induced arthritis (CIA), and in fact, even increasing the number of MSCs could not reduce affected paw swelling in the respective CIA mice due to the high level of proinflammatory cytokines, especially TNF-α (Djouad et al., 2005). In contrast, other *in vivo* studies of MSC effects on CIA indicated that MSCs can effectively inhibit CIA inflammation and joint pathology (Park et al., 2017; Sun Y. et al., 2017). Indeed, preliminary evidence for MSC efficacy has been reported in some RA patients (Alvaro-Gracia et al., 2017). MSCs could also suppress autophagy of activated T cells induced by respiratory mitochondrial metabolism in SLE patients (Chen et al., 2016) and, consequently, reduce T cell apoptosis and maybe play a crucial role in SLE treatment.

Overall, these findings strongly suggest a crucial therapeutic role for MSCs in regulating the proliferation and functional activation of lymphocytes and other immune cells in chronic inflammatory disease. Nevertheless, the relative numbers of MSCs and the proinflammatory cytokine environment may profoundly affect the immunoregulatory effect of MSC therapy.

## Genetic Factors Contributing to Msc Dysfunction in Sle

SLE is a heterogeneous autoimmune disease with clinical manifestations ranging from butterfly erythema and mild arthritis to severe lupus nephritis and lupus encephalopathy (Marion and Postlethwaite, 2014; Fanouriakis et al., 2019). SLE patients often have familial association, for example, monozygotic twins and siblings with a family history are more likely to suffer with lupus compared to siblings in families with no history of systemic autoimmune diseases (Cooper et al., 1999). Genome wide association studies (GWAS) have identified many genetic loci that associate with lupus (Sanchez et al., 2011; Bentham et al., 2015). Together the familial associations and GWAS indicate that SLE is a heterogeneous disease with strong, but complicated genetic background. Given that MSCs from SLE patients have dysfunctional immunomodulatory effect *in vitro*, genetic factors that contribute to, albeit heterogeneous, development of SLE may contribute to the dysfunction of autologous MSCs from SLE patients.

The human leukocyte antigen (HLA) complex encodes the major histocompatibility complex (MHC) proteins that regulate the immune system in humans (Bodis et al., 2018). Autoimmune diseases such as SLE, RA, ankylosing spondylitis (AS), and Behcet’s disease (BD) all have known associations with particular HLA alleles. SLE was found to have significant association with HLA-DMA and DMB alleles (Yen et al., 1999), but neither DMA nor DMB was correlated with disease activity. RA susceptibility is linked to HLA-DRB1 alleles (Raychaudhuri et al., 2012), and there is a strong linkage between ankylosing spondylitis (AS) and HLA-B27 (Brewerton et al., 1973). BD has a relatively strong correlation with HLA-B51 (Ohno et al., 1982).

HLA-G molecules are mainly expressed in human placental tissue (Curigliano et al., 2013). HLA-G generates seven alternative mRNAs encoding four membrane-bound isoforms (mHLA-G: HLA-G1, G2, G3, and G4) on the cellular surface and three soluble HLA-Gs (sHLA-G: HLA-G5, G6, and G7). HLA-G5 is one of the HLA-G family of non-classical MHC class I molecules that is secreted by MSCs. HLA-G5 was found to be critical for the immunomodulatory function of MSCs by inhibiting reactivity and cytolytic function of alloreactive T cells *in vitro* (Riteau et al., 2001; Selmani et al., 2008). The HLA-G5 alloprotective activity of the MSCs was mediated both by cell contact and soluble HLA-G5, and HLA-G5 secretion was enhanced by cell-cell contact between alloreactive T cells and MSCs. HLA-G5 functions in the initial cell contact between MSCs and stimulated, alloreactive T cells and contributes to the suppression of T cell proliferation and subsequent T cell differentiation toward Tregs (Selmani et al., 2008). HLA-G5 expression by MSCs was enhanced by IL-10 in a dose-dependent relationship. The latter finding is important since IL-10 is increased in SLE patients compared to healthy individuals. The association between IL-10 and HLA-G5 secretion by MSCs notwithstanding, BM-MSCs from SLE patients have a proinflammatory and senescence-associated phenotype mediated by a mitochondrial antiviral signaling protein (MAVS) that induces an IFN-β feedback loop (Gao et al., 2017). MAVS, IFN-β promoter stimulator protein 1, was significantly increased in SLE MSCs as were IFN-β-induced messenger RNAs. Notably, silencing of MAVS could downregulate IFN-β, p53, and p16 proteins and alter cytokine production in SLE MSCs. This newly identified pathway may provide critical insight about cellular mechanism that contribute to lupus autoimmunity and, as such, may define new potential therapeutic targets.

## Senescence-Associated Phenotype of Msc in Sle

BM-MSCs are gradually gaining attention because of their multidirectional differentiation potential that in turn may offer broad application prospects in clinical treatment of autoimmune diseases (Munir and McGettrick, 2015) and regenerative medicine (Mahla, 2016). However, MSCs from SLE patients possess a very limited proliferation potential *in vitro* and present a morphological appearance of senescence characterized by inflated volume, deeply stained nucleolus, and disordered cytoskeletal organization (Gao et al., 2017; Ji et al., 2017). The *in vitro* proliferative rate is decreased, and the proportion of apoptotic cells increased with MSCs from SLE patients compared with those from healthy, normal individuals.

MSCs from SLE patients also exhibit impaired capabilities for differentiation, migration, and immune regulation (Gao et al., 2017; Gu et al., 2016). MSCs from SLE patients have abnormalities in F-actin cytoskeleton accompanied by increased levels of intracellular reactive oxygen species (ROS) and MAVS, when compared to MSCs from normal, healthy individuals (Gao et al., 2017). The endoplasmic reticulum stress response (ERS) is involved in the senescence of MSCs from SLE patients and accounts for the dilated, distorted, and swollen morphology of SLE patient MSCs (Gu et al., 2015) detected by electron microscopy. The endoplasmic reticulum (ER) is an intracellular organelle that performs essential cellular functions including protein synthesis, post-translational modification, and protein folding (Ma and Hendershot, 2004). The endoplasmic reticulum stress response (ERS) occurs when chaperone proteins in the ER perceive and respond to abnormalities in normal ER function, such as protein folding (Ma and Hendershot, 2004). The ERS induces apoptosis and autophagy if the conditions that initiated the ERS are not resolved. However, the mechanisms that control the abnormal phenotype(s) of MSCs in SLE patients, including increased senescence and apoptosis, remain incompletely understood.

MSCs from SLE patients have elevated MAVS, TGF-β, ROS, telomerase activity, DNA damage, and increase expression of senescence associated genes that block the cell cycle (Nie et al., 2010; Gao et al., 2017). MSCs from SLE patients also have up-regulated immunoregulatory factors such as TGF-β, IDO-1, and LIF (Shi et al., 2014; Ji et al., 2017). MAVS is the only adaptor protein between retinoic acid-inducible gene I (RIG-I)-like receptors (RLRs) and NF-κB and the downstream IRF-3/7 signaling pathways (Vazquez and Horner, 2015). Thus, MAVS plays an indispensable role in the innate immune signaling pathway and induces IFN expression, especially IFN-β. A MAVS-IFN-β positive feedback loop essentially provides feed-forward stimulation for increased IFN-β in MSCs from SLE patients. Further, MSCs from SLE patients exhibited proinflammatory and aging features mediated by ROS induced as a consequence of the MAVS-IFN-β positive feedback loop (Shi et al., 2014; Gao et al., 2017; Ji et al., 2017). Growth restriction, enhanced β-gal activity, and impaired migration capacity are also consequences of the MAVS-IFN-β positive feedback loop. IFN-β triggers DNA signaling pathways by inducing chemical modification of p53, ROS that affects the transcriptional activity and function of P53, all of which interfere with the tumor inhibition pathways controlled by p53 that also trigger cellular aging and senescence (Moiseeva et al., 2006). Senescence-associated secretory phenotype–related gene expression, including IL-6, IL-8, and granulocyte–macrophage colony-stimulating factor (GM-CSF), is significantly increased in MSCs from SLE patients (Gao et al., 2017). In contrast, Bcl-2, which is important for inhibiting apoptosis (Li X. et al., 2012), is markedly reduced in SLE patient MSCs. Furthermore, abnormal activation of several signaling pathways including JAK-STAT, p53/p21, PTEN/Akt, PI3K/Akt, and Wnt/beta-catenin (Gu et al., 2014; Chen et al., 2015; Tan et al., 2015; Ji et al., 2017) are involved in development of the senescence phenotype in SLE patient MSCs. Inhibition or knockout of the expression of these pathways could reverse the senescent phenotype of MSCs from SLE patients, upregulate immunomodulatory cytokines such as TGF-β and IL-10, and downregulate proinflammatory cytokines, such as IFN-β, IL-17, and IL-6 (Tan et al., 2015; Gao et al., 2017). Thus, we speculated that the senescence of MSCs might be both a part of or an indicator for regulatory abnormalities in SLE patients, which may be related to the underlying SLE pathogenesis. Of note, IFN-β has a higher affinity to the type I IFN receptor than IFN-α (Schreiber and Piehler, 2015).

## Abnormal Inflammatory Niche in Sle

Autoimmune diseases create microenvironments of chronic inflammation as a consequence of immunological dysregulation that leads to excessive innate and adaptive immune stimulation. As a classic model systemic autoimmune disease, SLE can be characterized by the loss of peripheral immune tolerance, increased lymphocyte numbers and activation, and other immune cell activation, and autoantibody production, all of which contribute to pathogenic chronic inflammation (Marion and Postlethwaite, 2014). The homeostatic regulatory balance that generally prevents and controls autoimmunity is lost. Key among the regulatory elements lost are Tregs. Tregs function by downregulating the activation and proliferation of effector T cells (Li and Rudensky, 2016), and critical to SLE, Treg numbers are decreased in SLE patients, especially in active disease (Tselios et al., 2014).

In addition to inhibiting the secretion of proinflammatory cytokines, MSCs can also induce naïve CD4^+^T cells to differentiate into Tregs (Luz-Crawford et al., 2013; Wang et al., 2017a), indicating that allogeneic MSC transplantation may be able to restore the balance between Treg and Th in SLE patients. Working against that potential are antigen presenting cells, particularly plasmacytoid dendritic cells (pDC), that are the primary producers of IFN-α (Swiecki and Colonna, 2015). In SLE patients, pDC drive the differentiation of immature B cells to plasmablasts but fail to induce Bregs, and this compromised cross-talk with pDC and B cells has been associated with increased production of IFN-α (Menon et al., 2016). Additionally, the proinflammatory effect of IFN-α will not only promote T cell activation but will also stimulate more differentiation of CD4^+^ T cells to become effector cells, Tfh, Th1, and Th17, rather than iTregs further tipping the balance away from the immunosuppressive function of Tregs and loss of peripheral tolerance in SLE patients (Yan et al., 2008; Golding et al., 2010; Ambrosi et al., 2012).

The immunophenotype and immunoregulatory function of MSCs may be altered by microenvironments as a consequence of the local pro-inflammatory cytokine milieu (Djouad et al., 2005). For example, in SLE, aberrant accumulation and activation of immune cells and overexpression proinflammatory cytokines causes pivotal change in MSCs. The immunomodulatory function of MSCs stimulated with both IL-1β and TNF-α was pro-inflammatory and enhanced CD4^+^ T cell proliferation and differentiation to Th effector cell subsets rather than the anti-inflammatory immunomodulatory function of MSCs not stimulated with TNF-α and IL-1β (Dorraji et al., 2018). In fact, the role of TNF-α in MSCs remains controversial as TNF-α may exert different effects on MSC migration under different conditions. Normally, TNF-α upregulates MSC migration by activation of the NF-κB signaling pathway via IKK-2 (Haasters et al., 2013), a key regulatory enzyme of the NF-κB pathway. However, the significantly increased TNF-α in SLE patient serum inhibited the migration capacity of MSCs revealing what seems to be an impaired phenotype in the TNF-α-dependent migration of bone marrow-derived MSCs from SLE patients (Geng et al., 2014).

IDO, mainly secreted by DCs and macrophages, is an enzyme that mediates tryptophan degradation into immunosuppressive metabolites. Lipopolysaccharide and cytokines especially, IFN-γ, can induce the expression of IDO during inflammation or infection (Pallotta et al., 2011). Studies have demonstrated that IDO plays an indispensable role in allogenic MSC-mediated inhibition of T cell proliferation in lupus patients, which could be enhanced by IFN-γ (Wang et al., 2014). Intriguingly, MSCs from active SLE patients exhibited defective IDO production under IFN-γ stimulation (Wang et al., 2014). Consequently, modulation of IDO activity might be a novel therapeutic way to restore the defective properties of SLE patient MSCs. In contrast, mouse MSCs require nitric oxide synthase (NOS) instead of IDO to catalyze production of NO to mediate their immunosuppressive function (Sato et al., 2007; Ren et al., 2008). There are three isozyme subtypes of NOS, including neuronal nitric oxide synthase (nNOS) and endothelial nitric oxide synthase (eNOS) expressed under normal conditions, and inducible nitric oxide (iNOS) induced by injury (Forstermann and Sessa, 2012). MSCs were able to inhibit Tfh cells in lupus-prone mice by producing NO, and iNOS was an important mediator in the process since _*L*_-NMMA, a specific inhibitor of iNOS, could partially restore the generation of Tfh cells inhibited by MSCs *in vitro* (Sato et al., 2007; Zhang et al., 2017).

There is another reason why transplanted autologous MSCs may fail to suppress the excessive and damaging immune reactions in SLE patients. The number of autologous immunosuppressive MSCs present in relevant organs and tissues subject to chronic inflammation after autologous transplantaion may be too low to exert an efficient immunosuppressive effect. Compared with MSCs from healthy individuals, MSCs from SLE patients are morphologically biased toward senescent cells with reduced proliferative and migratory capabilities (Geng et al., 2014; Gao et al., 2017). Whether this abnormality is an inherent MSC defect alone or in addition to effects from drug treatment requires further research. As discussed above, previous studies showed that inhibition or reversal of the MSC aging-associated genes or signaling pathways could partially or fully reverse the senescent phenotype and immunoregulatory function of SLE patient MSCs (Gu et al., 2014; Chen et al., 2015; Tan et al., 2015; Ji et al., 2017). Consequently, reversing MSC senescence may allow autologous MSCs to be an effective therapy for SLE.

## Advantages and Prospects of Treating Sle With Mscs

SLE is a systemic autoimmune disorder involving a multitude of autoantibodies that are produced by over-activated B cells that circulate in peripheral blood and deposit in organs (Marion and Postlethwaite, 2014). The autoantibodies are produced by B cells activated in germinal centers to produce isotype-switched, somatically mutated IgG autoantibodies most notably specific for nuclear antigens but other autoantigens as well. SLE is more prevalent in women of childbearing age and shows a significant gender bias with a male to female ratio of 1:9, although men and children tend to have more severe disease (Bernatsky et al., 2006; Aggarwal and Srivastava, 2015; Hwang et al., 2015). Most SLE patients show a chronic remission-relapse course except for a small number of patients that can achieve long-term remission (Fanouriakis et al., 2019). SLE is a potentially fatal autoimmune disease that can affect multiple tissues and organ with lupus nephritis being one of the most common and severe complications (Marion and Postlethwaite, 2014). Among all the complications, renal involvement carries substantial mortality and morbidity.

Treatment of SLE is challenging because of clinical heterogeneity and unpredictable disease flares. The current guideline for treating moderate to severe lupus nephritis is a two-stage treatment regimen including an initial induction phase and a prolonged maintenance phase (Fanouriakis et al., 2019). Induction therapy with intensive immunosuppressive agents, for example, high-dose methylprednisolone combined with cyclophosphamide (CTX) intravenous infusion, is adopted at the initial stage to control autoantibody production and lymphocyte activation, restore organ function and inhibit tissue damage (Austin et al., 1986; Grootscholten et al., 2006). To consolidate disease remittance and reduce recurrence, long-term maintenance treatment with less intense and moderate side effects such as low-dose prednisone and mycophenolate mofetil (MMF) is recommended (Contreras et al., 2004; Ruiz-Irastorza et al., 2010). Although most lupus patients respond well to the conventional treatment of steroid and immunosuppressive agents such as CTX, tacrolimus, and MMF, there are remarkable and potentially serious side effects associated with each including infection, metabolic disorders, ischemic osteonecrosis, gastrointestinal adverse reactions, liver and renal toxicity, gonadal inhibition, and myelosuppression (Kamanamool et al., 2010; Ishii et al., 2015; Kishi et al., 2018; Fanouriakis et al., 2019). Conventional therapy usually requires the use of multiple immunosuppressive agents for several years or even for the lifetime of a patient. Long-term complex prescriptions and tapering methods, regular follow-up and routine blood tests not only plague the patient’s daily life, but also aggravate the patient’s financial and psychological burdens (Barber and Clarke, 2017; Zhao et al., 2018). Even worse are refractory patients who fail to response to conventional therapy and have persistently active disease (Nikpour et al., 2009). Therefore, there is a strong, urgent need to develop a new treatment for SLE that not only can effectively control disease flares with acceptable side effects, but also reduce the patient’s burden for continuous medication and extend the follow-up time. B cell depletion therapies have engendered hope for availability and effectiveness of new biologics to treat, if not cure, lupus (Looney, 2010). Results from both the EXPLORER (Looney et al., 2010) and LUNAR (Rovin et al., 2012) clinical trials of the B cell targeting monoclonal antibody rituximab were disappointing since neither trial achieved the predetermined endpoint for success. Belimumab trials have been much more promising with significant improvement in immunologic parameters, but clinical disease improvement was still only moderate compared to placebo (Navarra et al., 2011; Manzi et al., 2012; Stohl et al., 2012). New approaches and new therapies are desperately needed for SLE. The more recent success of B cell depletion with anti-CD19 CAR-T (chimeric antigen receptor-T) cells in lupus-prone mice may offer an alternative, more successful approach for B cell depletion therapy for SLE in humans (Kansal et al., 2019). MSC may be the much-needed new approach to therapy.

For patients who respond poorly to conventional therapy, MSC therapy has shown satisfactory efficacy with acceptable treatment-related adverse events (Wang et al., 2017b; Barbado et al., 2018; Liang et al., 2018). Data from North and South American transplantation centers have indicated that the 3 years MSC transplantation-related mortality (TRM) was no more than 5% (Pasquini et al., 2012), while in a Chinese long-term retrospective study, the TRM was 0.2% (1/404) (Liang et al., 2018).

In recent years, various countries or regions have attached great importance to stem cell research and clinical translation. The number of clinical trials for stem cell-based therapies for autoimmune diseases registered in *www.clinicaltrials.gov* website reached 212 cases worldwide as of July 4, 2019. Moreover, there are 14 different stem cell-associated products approved for therapy^1^, most of which are MSCs or HSCs.

Anecdotal case reports have reported that total remission can be achieved after HSC transplantation in refractory SLE patients with severe disease and who were resistant to conventional therapies (Traynor and Burt, 1999; Rosen et al., 2000). In a 5 year follow-up study, most of the refractory SLE patients who underwent autologous HSC infusion, after immunoablation and depletion of mononuclear cells, attained durable clinical and serological remission (Burt et al., 2018). In another clinical study, autologous HSC transfusion also restored Treg numbers and immunosuppressive function in SLE patients (Zhang et al., 2009). Autologous HSC transfusion in conjunction with non-myeloablative immunoablation with CTX, rituximab, and thymoglobulin (rATG) may be therapeutically effective in SLE since the autoreactive immune cell clones are eliminated, and the reconstituted immune competent cells develop with normal, effective self-tolerance. The most common adverse events in the treatment with HSC are fever, infection, and infusion reactions, but most adverse events were determined to be unrelated to infusion (Liang et al., 2018). The success of autologous HSC therapies has encouraged a possible therapeutic use of MSCs to treat SLE patients.

MSCs are non-hematopoietic, multipotent progenitor stem cells that possess immunomodulatory capabilities. Several clinical studies have been performed to evaluate the potential clinical efficacy for MSC transplantation as an alternative therapeutic approach to the current pharmacologic therapy for SLE. Results from those studies indicate that MSC transplantation is a safe and effective therapy (Munir and McGettrick, 2015; Squillaro et al., 2016; Wang et al., 2017b) that can ameliorate multiorgan injuries and induce long-term disease remission in active and refractory SLE patients (Liang et al., 2010; Deng et al., 2017; Barbado et al., 2018). MSCs were able to re-establish the defective osteoblastic niche in lupus-prone mice and effectively reverse multiorgan dysfunction, especially glomerulonephritis, in mice and patients compared with CTX (Sun et al., 2009; Choi et al., 2016). Unlike anti-CD20 and TNF inhibitor biological agents, MSC could restore the ratio of Treg/Tfh cells in CIA mice (Sun Y. et al., 2017). Finally, experiments in mice have evaluated the effect of combination therapy with MSCs and five kinds of drugs including prednisone, dexamethasone, cyclosporine A, mycophenolate mofetil, and rapamycin for their effects on T cell subpopulations. The results indicated that MSCs could enhance the anti-inflammatory effects of the drugs and attenuate the cytotoxic side effects of the immunosuppressants (Hajkova et al., 2017). Hence the combination of MSCs and immunosuppressants may become a more ideal therapeutic strategy to treat autoimmune diseases, especially SLE, compared to pharmacologic immunosuppression alone.

Our review shows that, although allogeneic MSCs are promising candidates for treating SLE, autologous MSCs may not be therapeutically useful because of their defects in both immunomodulatory function and regenerative characteristics. MSCs from SLE patients present a morphological appearance of senescence with impaired capabilities of differentiation, migration and immune regulation (Gu et al., 2016; Gao et al., 2017; Ji et al., 2017). B cells contribute to pathogenesis in SLE through both autoantibody-dependent and autoantibody-independent mechanisms. Abnormally activated B cells in SLE patients affect the function of MSCs, and depletion of B cells may help to restore the potential immunosuppressive of autologous MSCs. Notably, previous studies have shown that MSCs are capable of inhibiting the proliferation and differentiation of B cells (Corcione et al., 2006) and hence may have a promising efficacy in treating SLE. On the other hand, MSCs could enhance proliferation and differentiation into immunoglobulin-secreting cells of naïve and transitional B lymphocytes from SLE patients *in vitro* (Traggiai et al., 2008) raising concerns regarding the therapeutic use of MSC to suppress B lymphocytes. These results suggest caution in considering and monitoring MSC therapy to treat SLE. Further studies are needed to uncover the underlying regulation between MSC and immune cells and how those effects may affect disease in SLE patients.

## Conclusion

**FIGURE 3:**
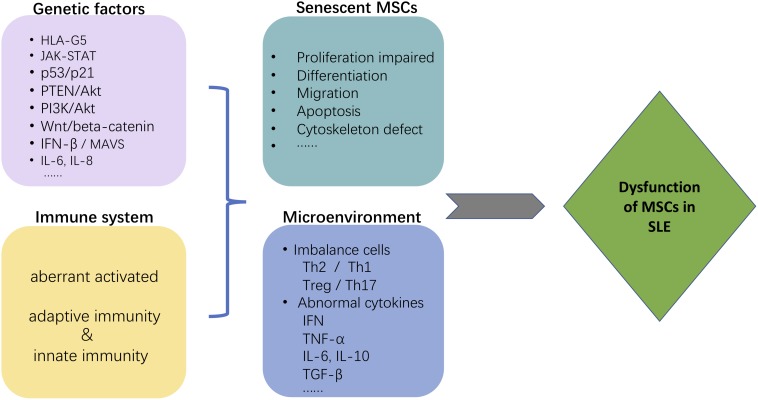
Possible mechanism that may contribute to MSC dysfunction in SLE. The figure depicts several hypothetical mechanisms to explain why MSCs are defective in SLE patients. Both genetic factors and the immune system environment, particularly pro-inflammatory, are expected to contribute to the immunosuppressive dysfunction of MSCs from SLE patients. The morphological changes associated with aging of MSCs from SLE patients are the consequence of several senescence-associated genes and signaling pathways. The proinflammatory niche created by immune system dysfunction in SLE synergistically contributes to the abnormalities in MSC.

Likely, no single factor can account for the intricate mechanisms of the MSCs-mediated immunosuppressive effect on autoimmune diseases. There are several hypothetical mechanisms why MSCs from SLE patients appear defective (see Figure 3). GWAS have indicated that autoimmune diseases, including SLE, are associated with numerous, heterogeneous genetic loci. MSCs from SLE patients are characterized by morphological and phenotypic changes associated with aging. These include functional changes modulated by expression of several senescence-associated genes and signaling pathways that decrease proliferative potential. Likely, the proinflammatory microenvironment in patients with lupus alters the immunosuppressive potential of MSCs from those patients. Similar results were found in patients with other inflammatory diseases such as abdominal aortic aneurysm (AAA). MSCs isolated from human AAA wall display a dysregulated immunosuppressive effect on peripheral blood mononuclear cells (PBMCs) proliferation (Ciavarella et al., 2015).

In conclusion, murine models and clinical trials have produced evidence for the therapeutic potential of MSCs for SLE. Our review shows that although allogeneic MSCs are promising candidates for treating SLE, autologous MSCs may not be eligible to treat SLE patients because of their defective immunomodulatory function and poor regenerative characteristics. Moreover, whether the immunological rejection of allogeneic stem cell transplantation will influence the efficacy of MSC therapies or have long-term effects on recipients is not known. If the causes of MSC dysfunction for MSCs from SLE patients can be better understood in the future, maybe modification or transformation of SLE patient MSCs to restore immunosuppressive and regenerative function can yield a therapeutically beneficial treatment. In fact, recent research has indicated that MSCs transfected with an etanercept-encoding vector can successfully produce the drug *in vitro* and had superior suppressive effects in CIA mice compared to non-modified MSCs (Park et al., 2017). Prospects for MSCs as immunosuppressive therapy in other rheumatic diseases are being and should continue to be explored. However, only through further research and clinical trials can we completely resolve this mystery of why MSCs are defective in SLE patients. Studying the mechanism of MSC defects in SLE patients can provide new ideas for the pathogenesis of SLE, and provide a new theoretical corroborate for cellular therapy that may be of great significance for clinical application in rheumatic diseases.

## Author Contributions

R-JC wrote the original manuscript. A-JX and Y-HL organized sections of the manuscript. S-YP and Q-PZ prepared the figures. YZ, YL, and TM revised the manuscript.

## Conflict of Interest

The authors declare that the research was conducted in the absence of any commercial or financial relationships that could be construed as a potential conflict of interest.

## Footnotes

1 www.clinicaltrials.gov
